# A Role for MK2 in Enhancing Neutrophil-Derived ROS Production and Aggravating Liver Ischemia/Reperfusion Injury

**DOI:** 10.3389/fimmu.2018.02610

**Published:** 2018-11-13

**Authors:** Lei Sun, Qiong Wu, Yunjuan Nie, Ni Cheng, Rui Wang, Gang Wang, Dan Zhang, Huiqiong He, Richard D. Ye, Feng Qian

**Affiliations:** ^1^Engineering Research Center of Cell & Therapeutic Antibody, Ministry of Education, School of Pharmacy, Shanghai Jiao Tong University, Shanghai, China; ^2^Anhui Province Key Laboratory of Translational Cancer Research, Bengbu Medical College, Bengbu, China; ^3^Department of Pharmacology, University of Illinois College of Medicine, Chicago, IL, United States; ^4^Jiangsu Center for the Collaboration and Innovation of Cancer Biotherapy, Cancer Institute, Xuzhou Medical University, Xuzhou, China; ^5^Institute of Chinese Medical Sciences, University of Macau, Macau, China

**Keywords:** MK2, ROS, p47^phox^, neutrophils, hepatic ischemia/reperfusion injury

## Abstract

Increased inflammatory responses and enhanced reactive oxygen species contribute to hepatic ischemia/reperfusion (I/R) injury, however the modulatory mechanisms haven't been completely unveiled. Here, we report that genetic deficiency of MAPK-activated protein kinase 2 (MK2) protected against hepatic I/R injury and decreased hepatic neutrophil accumulation in MK2^−/−^ mice. Depletion of neutrophil attenuated hepatic I/R injury in wide type mice. In response to C5a stimulation, MK2^−/−^ neutrophils generated less superoxide in which both NADPH oxidase activation and p47^phox^ phosphorylation were decreased. Furthermore, Ser329 of p47^phox^ was identified for enhancement of superoxide production. The Ser329 phosphorylation was reduced in MK2^−/−^ neutrophils. To determine whether MK2 modulates hepatic I/R injury via activating neutrophils, we generated myeloid-specific MK2 deletion mice (MK2^Lyz2−KO^) and liver I/R injury was reduced in MK2^Lyz2−KO^ mice. Our results indicate that MK2 augments hepatic I/R injury and induces ROS production with increased p47^phox^ phosphorylation and MK2 is a potential drug target for treating hepatic I/R injury.

## Introduction

Hepatic ischemia/reperfusion (I/R) is a pathophysiologic process that can be triggered by liver transplantation, elective liver surgery, toxic liver injury, and hepatic sinusoidal obstruction syndrome (1). Hepatic I/R injury is induced by reperfusion of blood flow and hypoxia accentuation in ischemic tissues, which leading to traumatic hemorrhagic shock, live damage, and graft dysfunction (2). Despite the recent improvements in liver preservation and surgical techniques, hepatic I/R injury remains an important clinical problem during liver surgery. Therefore, it is of importance to comprehensively understand the mechanisms of hepatic I/R and develop novel therapeutic approaches.

Hepatic I/R injury is a complex pathophysiological process. Initial phase of hepatic I/R injury is associated with oxidative stress, anaerobic metabolism, and calcium overload. At late phase, an increase in the cellular damage and, immune response contribute to the progression of parenchymal injury (3). Overall, reactive oxygen species (ROS), pro-inflammatory cytokines and chemokines as well as Kupffer cells (KCs), neutrophils and lymphocytes are involved in the process and are closely interlocked (4–6).

Neutrophils have a pivotal role in the pathogenesis of hepatic I/R injury (7). During hepatic I/R injury, neutrophils are first activated by chemokines, and migrate across the endothelium to the hepatocytes (7, 8). Then neutrophils further damage endothelial cells and destroy the integrity of the microvasculature through release of ROS, proteinases (cathepsin G, granulocytes elastase) and cationic peptides. Inhibition of neutrophil infiltration can protect against hepatocellular injury following I/R (7). However, the molecular mechanisms and regulation of neutrophils-derived ROS production have not been defined.

MAPK-activating protein kinase 2 (MK2) is a major effector serine/threonine-protein kinase downstream of p38-alpha (p38α) MAPK (9, 10). Once activated, MK2 phosphorylates many substrates and is implicated in many cellular processes including stress and inflammatory responses, cytoskeleton modulation, nuclear export, gene expression and cell proliferation (10, 11). MK2 plays a vital role in several diseases, such as cancer (12, 13), neurodegenerative disease (14), and inflammatory diseases (10, 15). Recently, Ashraf et al. reported that p38 MAPK activity is increased upon reperfusion and p38 MAPK inhibition prevents severe functional impairment caused by I/R (16). However, It has not been demonstrated how p38 MAPK modulates hepatic I/R and whether MK2 is a critical gene in modulation of hepatic I/R injury.

Using a mouse model of hepatic I/R injury, we found that complete MK2 deficiency markedly alleviated liver damage, serum alanine aminotransferase levels, intrahepatic macrophage/neutrophil trafficking, and pro-infiammatory cytokine production. Depletion of neutrophils *in vivo* reduced hepatic I/R injury and MK2 was required for NADPH oxidase activation and superoxide production. We also identify p47^phox^ as a substrate of MK2, in which MK2 phosphorylated serine 329 residue of p47^phox^ and this modification was required for NADPH oxidase activation. Furthermore, we proved that conditional depletion of MK2 in neutrophils also protected against hepatic I/R injury. Collectively, our findings reveal a critical role of MK2 in promoting ROS production and accentuating hepatic I/R injury.

## Results

### Genetic MK2 deficiency alleviates liver injury caused by hepatic I/R in mice

**Figure 1:**
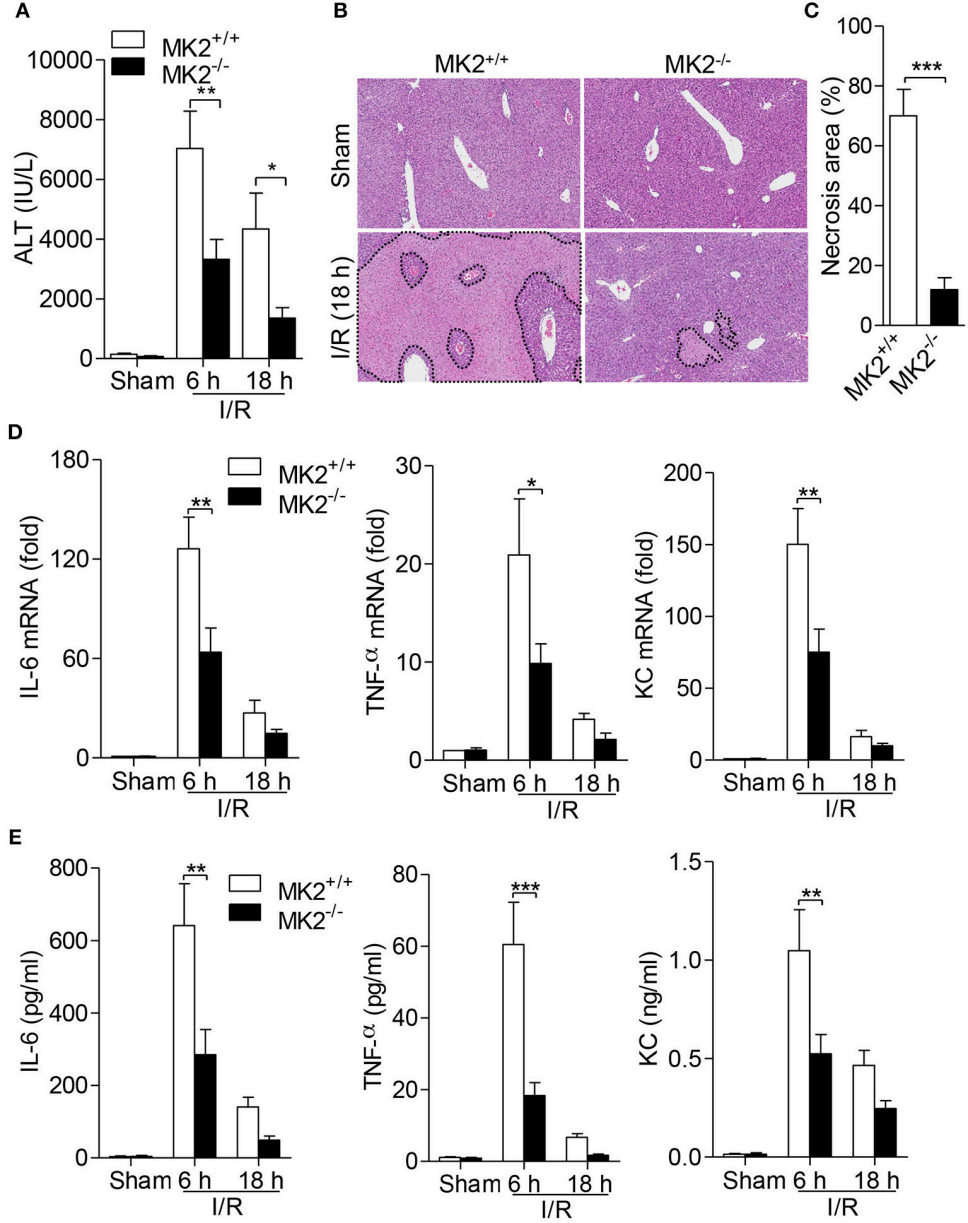
Genetic MK2 deficiency alleviated hepatic I/R injury and inflammatory cytokine production. MK2^+/+^ and MK2^−/−^ mice were subjected to 60 min of partial liver warm ischemia, followed by reperfusion for 6 and 18 h. **(A)** Mouse blood was collected and serum ALT was detected. **(B)** Hepatic I/R injury was evaluated by hematoxylin-and-eosin (H&E) staining of injury liver tissues. Original magnification × 100. **(C)** The necrosis area was quantified by using ImageJ software. **(D)** The mRNA levels of IL-6, TNF-α, and KC in injury livers were detected by qPCR. **(E)** The protein levels of IL-6, TNF-α, and KC in serum were detected by ELISA. The results are shown as means ± SEM. ^*^P < 0.05; ^**^P < 0.01, ^***^P < 0.001, based on 5 mice in each group.

MK2 plays a critical role in inflammation and cell proliferation (10), however its role in hepatic I/R injury remains unknown. To determine the effect of MK2 on hepatic I/R, we performed a hepatic I/R mouse model in genetic MK2 deficiency mice (referred to as MK2^−/−^ mice) with 60 min of partial liver warm ischemia followed by reperfusion for 6 and 18 h. Compared to that in MK2^+/+^ mice, the serum ALT levels in MK2^−/−^ mice were significantly reduced at 6 and 18 h (Figure 1A). Based on H&E staining, the liver damage was concordant with the change of serum ALT so that liver necrosis area was attenuated in MK2^−/−^ mice (Figure 1B). Image-Pro Plus software analysis revealed statistically significant decreased necrosis area in livers from MK2^−/−^ mice, compared to that from MK2^+/+^ mice (Figure 1C). Collectively, these results indicated that MK2 deficiency dramatically ameliorates liver damage during hepatic I/R injury.

### MK2 deficiency reduces inflammatory cytokine production

Given that inflammatory cytokines contribute to the occurrence and development of hepatic I/R injury (6), we further determined whether MK2 modulates cytokine production in hepatic I/R injury. Six hours after hepatic I/R injury, significant increases of IL-6, TNF-α and KC mRNA of liver were observed in MK2^+/+^ mice (Figures 1D,E). However, deletion of MK2 resulted in dramatic decrease of these cytokine at 6 h after I/R injury (Figure 1D). Similarly, compared with that in MK2^+/+^ mice, the levels of IL-6, TNF-α and KC in serum were remarkably attenuated in MK2^−/−^ mice at 6 h after hepatic I/R (Figure 1E). These results indicate that MK2 contributes to the hepatic I/R-induced increase of inflammatory cytokines in mice.

### MK2 is required for neutrophil infiltration during hepatic I/R injury

**Figure 2:**
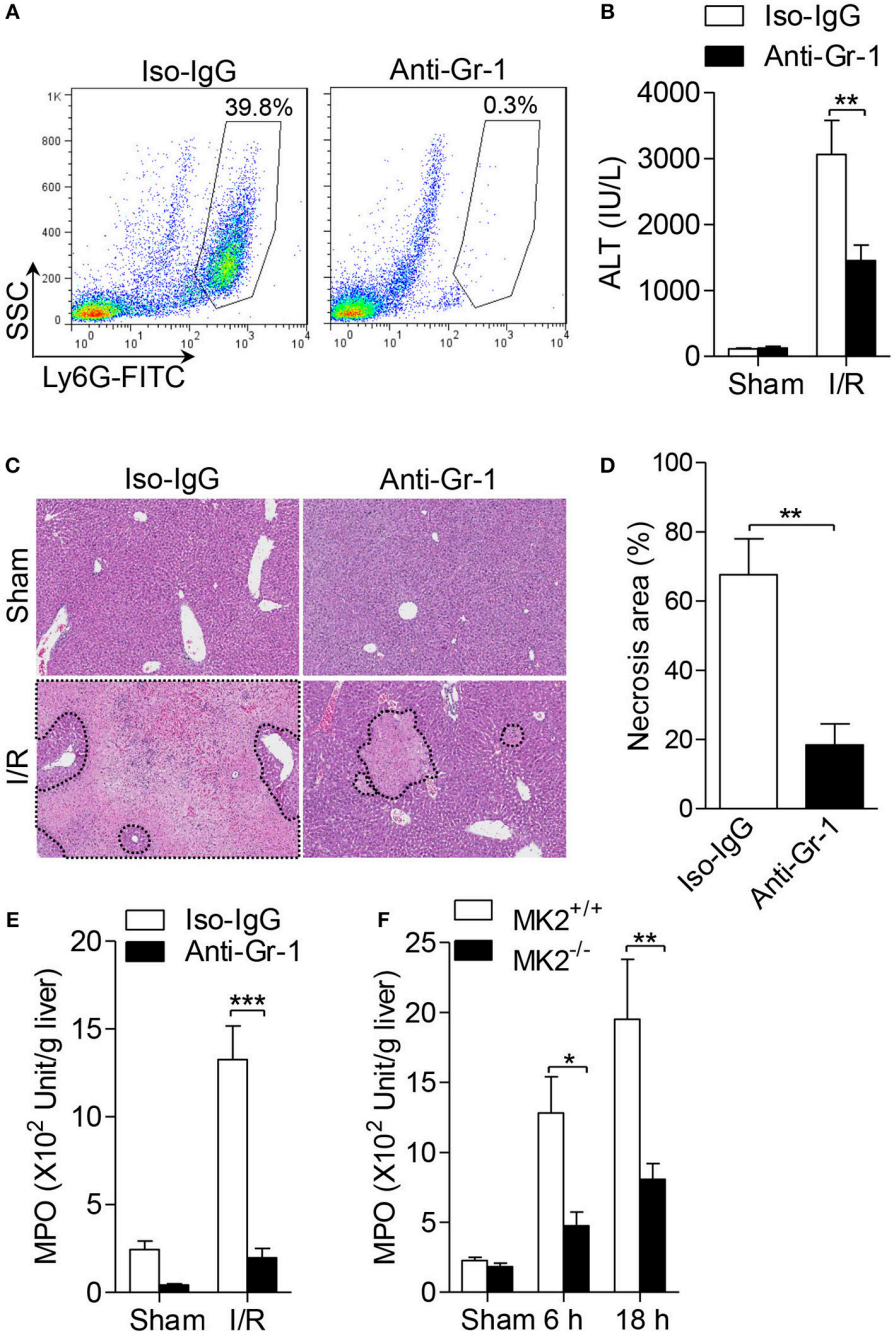
Genetic MK2 deficiency reduced neutrophil trafficking in hepatic I/R injury. **(A)** The wild type mice (MK2^+/+^) were intraperitoneally injected with anti-Gr-1 antibody (1A8), or isotype-matched IgG as control for 24 h, and then the percentage of neutrophils in peripheral blood were shown, as determined by anti-Ly6G-FITC FACS staining. **(B)** After injected with anti-Gr-1 antibody as in **(A)**, mice were subjected to hepatic ischemia for 60 min and reperfusion for 18 h. Serum ALT was detected for evaluation of liver injury. **(C)** Representative histological staining (H&E) of ischemic liver tissue was displayed. Original magnification × 100. **(D)** The necrosis area was quantified by ImageJ software. **(E)** The activities of MPO activity of injury liver tissues were detected. **(F)** MK2^+/+^ and MK2^−/−^ mice were subjected to hepatic I/R injury as in Figure 1, the activities of MPO in liver tissue extract were evaluated. The results are shown as means ± SEM. *P < 0.05; **P < 0.01, ***P < 0.001, based on 5 mice in each group.

Because neutrophil infiltration is associated with hepatic I/R injury, we firstly determined whether neutrophils contribute to hepatic I/R injury by depleting neutrophils with anti-Gr-1 antibody (1A8). As showed in Figure 2A, administration of anti-Gr-1 intraperitoneally (i.p.) effectively reduced neutrophil count in peripheral blood to 0.3%, compared to control mice receiving isotype-IgG which showed 39.8% of neutrophils in peripheral blood (Figure 2A). Then, mice received anti-Gr-1 antibody or an IgG control were subjected to hepatic I/R. As shown in Figure 2B, mice receiving anti-Gr-1 antibody displayed significantly reduced ALT in the serum. In the I/R groups, less necrotic areas were observed after neutrophil depletion based on histologic analysis and statistical analysis (Figures 2C,D). Additionally, to evaluate the infiltration and activation of neutrophils, we assessed the myeloperoxidase (MPO) activity. As shown in Figure 2E, mice receiving anti-Gr-1 antibody displayed a lower MPO activity. Furthermore, we investigated the role for MK2 in the accumulation of neutrophils during hepatic I/R injury. As showed in Figure 2F, increased MPO activity was significantly decreased after MK2 deletion during hepatic I/R injury (Figure 2F). Taken together, these results indicate that neutrophils are required for hepatic I/R injury and MK2 contributes to local accumulation of neutrophils during hepatic I/R injury.

### MK2 is required for neutrophil superoxide production

**Figure 3:**
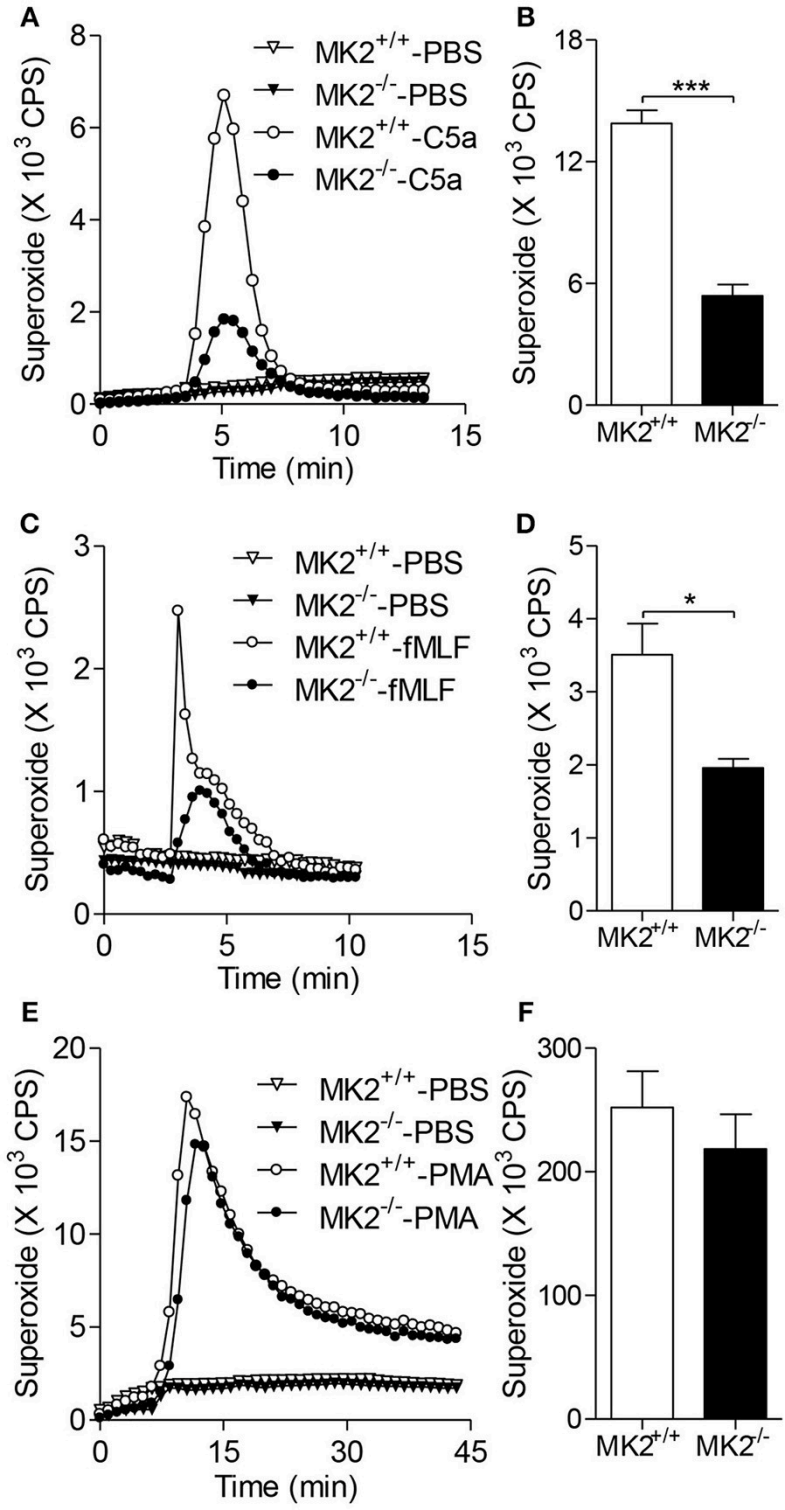
MK2 modulated neutrophil superoxide production. Neutrophils were isolated from mice and isoluminol-ECL was used to measure superoxide production. Representative tracing showing the production of superoxide (left) and cumulative superoxide production was quantified based the area under tracing line (right). The MK2^+/+^ and MK2^−/−^ neutrophils were stimulated with 100 nM of C5a or PBS **(A,B)**, 10 μM of fMLF or PBS **(C,D)**, and 200 ng/ml of PMA or PBS **(E,F)**. The results were presented as means ± SEM, *P < 0.05; ***P < 0.001, compared with similarly treated cells from MK2^+/+^ mice. The experiments were repeated at least 3 times.

It has been demonstrated that oxidant stress, especially the excessive reactive oxygen species (ROS), acts as one of the main modulators of hepatic I/R. Neutrophils are one of main sources of oxygen radicals in the post-ischemic liver, in which nicotinamide adenine dinucleotide phosphate (NADPH) oxidase is activated upon adhesion or by pro-inflammatory cytokines and the complement system (17). To determine the role of MK2 in oxygen radical production by neutrophils, we isolated polymorphonuclear neutrophils (PMN) from bone marrow of MK2^−/−^ and MK2^+/+^ mice. As shown in Figures 3A,C,E, the baseline of superoxide generation in neutrophils from MK2^−/−^ were not changed compared with MK2^+/+^ mice. Upon treatment with C5a, neutrophils from MK2^−/−^ mice generated significantly less superoxide compared to that from MK2^+/+^ mice (Figures 3A,B). Besides activated complement component, bacterial derived formylated peptide formyl-methionyl-leucyl-phenylalanine (fMLF) also tightly regulates the activation of the NAPDH oxidase in a receptor specific manner (18). Here, MK2^−/−^ neutrophils treated with fMLF showed significantly impaired superoxide production compared to that from MK2^+/+^ mice (Figures 3C,D). PMA is another NADPH oxidase agonist, which bypasses receptors and directly activates PKC. It is interesting that PMA-stimulated superoxide production showed no significant difference between neutrophils from MK2^+/+^ and MK2^−/−^ mice (Figures 3E,F). Collectively, these findings indicated that MK2 is involved in the regulation of chemoattractant receptor-dependent neutrophil NADPH oxidase activation and superoxide production.

### MK2 regulates AKT and P38 MAPK phosphorylation in neutrophils

**Figure 4:**
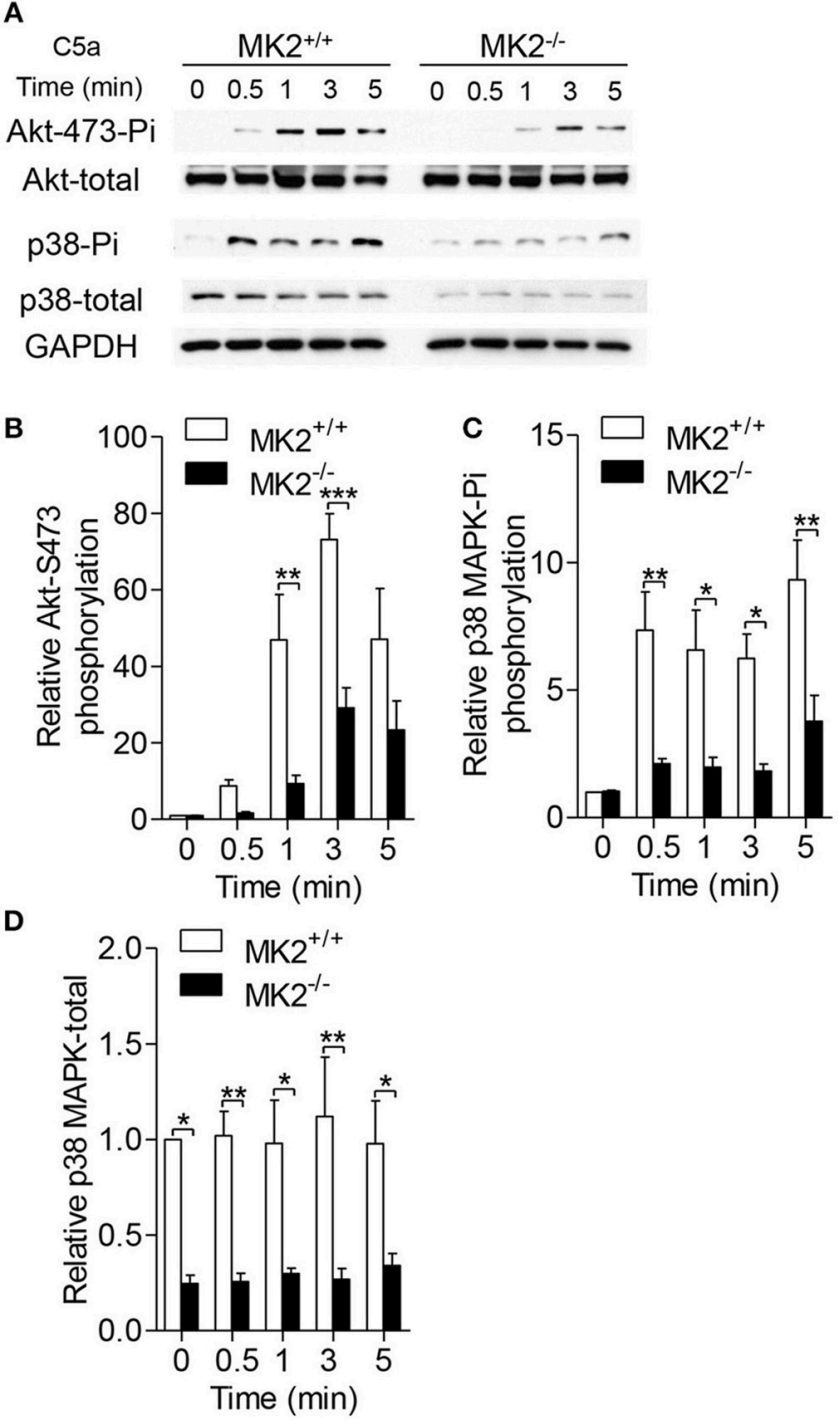
MK2 was required for AKT and p38 MAPK phosphorylation in neutrophils. **(A)** Neutrophils from MK2^+/+^ and MK2^−/−^ mice were challenged with C5a (100 nM) for indicated time. Phosphorylation of AKT (Ser473) and p38 MAPK was determined by western blotting using anti-phospho-antibodies against the phospho-AKT (Ser473), total AKT, phospho-p38 MAPK, and total p38 MAPK. **(B–D)** Densitometry analysis was conducted to determine the relative level of induced AKT phosphorylation, p38 MAPK phosphorylation, and total p38 MAPK. Data shown are means ± SEM from three independent experiments. *P < 0.05; **P < 0.01; ***P < 0.001.

Since several signaling pathways contribute to the activity of NADPH oxidase, we therefore reasoned whether MK2 has crosstalk with these signals. As shown in Figure 4A, neutrophils purified from the MK2^+/+^ and MK2^−/−^ mice were stimulated with 100 nM C5a for 5 min. Phosphorylation of AKT and p38 MAPK was determined by western blotting using respective antibodies. In response to C5a stimulation, the MK2^−/−^ neutrophils exhibited attenuated phosphorylation of AKT (Ser473) and p38 MAPK with unaltered kinetics, compared to MK2^+/+^ neutrophils (Figures 4A–C). In addition, the total level of p38 MAPK was also greatly decreased in MK2^−/−^ neutrophils (Figures 4A,D). Altogether, these findings suggest that MK2 is required for C5a-induced activation of AKT and p38 MAPK, two critical kinases for modulation of NADPH oxidase activation.

### MK2 directly phosphorylates P47^phox^ and regulates its membrane translocation

**Figure 5:**
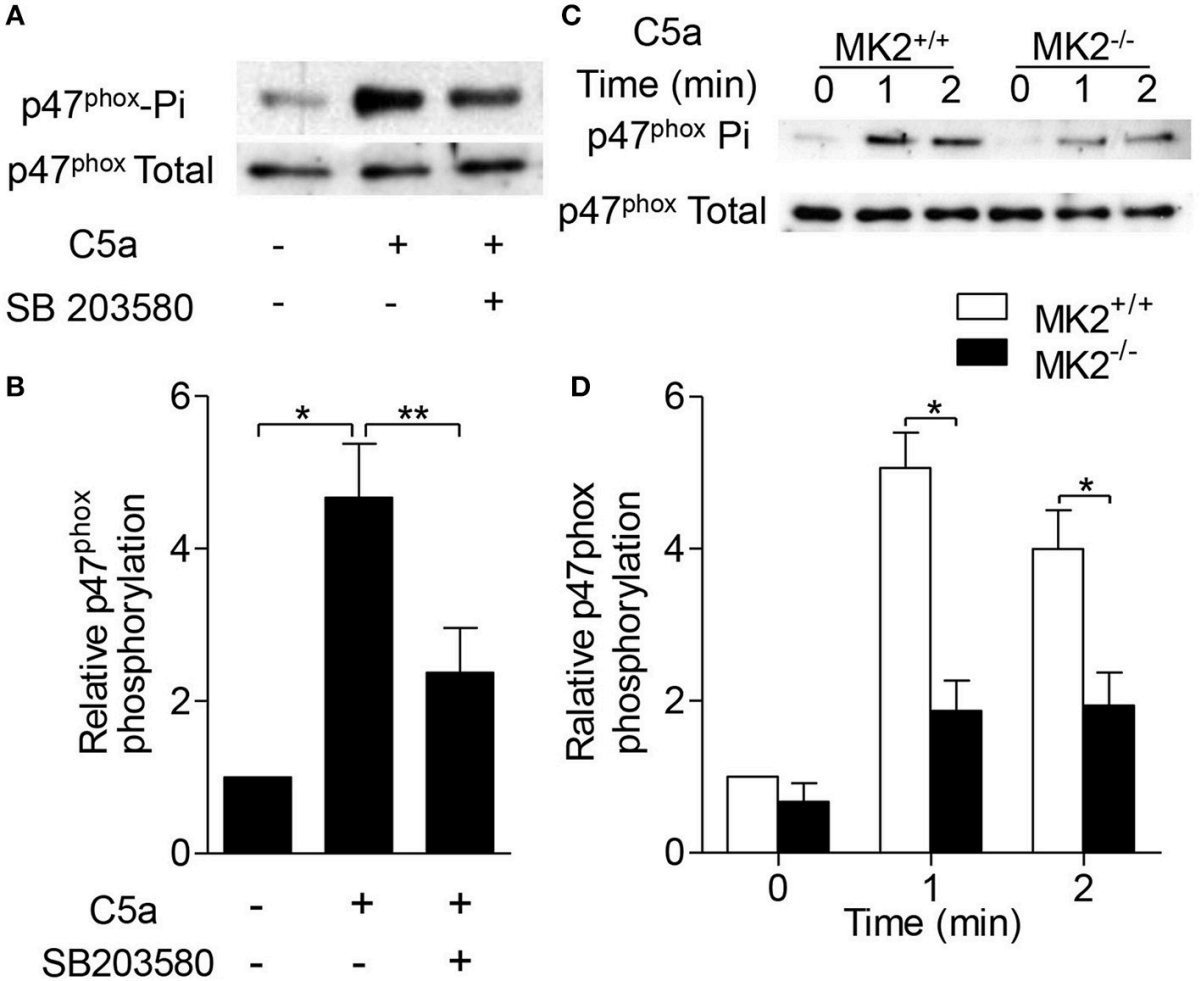
MK2 was essential for p47^phox^ phosphorylation in response to C5a stimulation. **(A)** Neutrophils from WT mice were pre-treated with SB203580 (3 μM) for 15 min, and then stimulated with C5a (100 nM) for 1 min. The phosphorylation of p47^phox^ was determined. **(B)** Densitometry analysis was conducted to determine the 47^phox^ phosphorylation. **(C)** Neutrophils from MK2^+/+^ and MK2^−/−^ mice were stimulated with C5a (100 nM) for 1 and 2 min. The phosphorylation (Phospho) of p47^phox^ was determined. **(D)** Densitometry analysis was conducted to determine 47^phox^ phosphorylation in MK2^+/+^ and MK2^−/−^ neutrophils. The results are shown as means ± SEM. ^*^P < 0.05; ^**^P < 0.01, based on three independent experiments.

**Figure 6:**
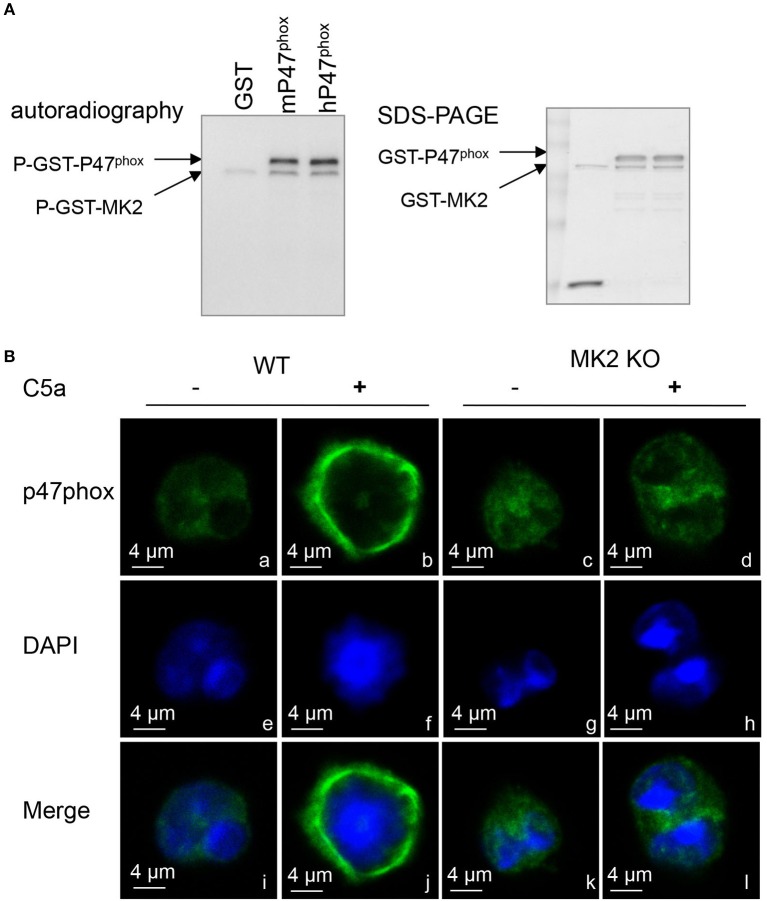
MK2 directly regulated p47^phox^ phosphorylation and membrane translocation. **(A)** Autoradiograph of *in vitro* kinase assay using full-length human p47^phox^ (hp47^phox^) or mouse p47^phox^ (mp47^phox^) fused to GST as substrates for GST-MK2. GST without mp47^phox^ was used as a negative control. Two phosphorylated bands were identified in the autoradiograph: a phosphorylated GST–p47^phox^, and an autophosphorylated GST-MK2. The loading levels of each protein were indicated in coomassie blue staining (right). **(B)** Neutrophils from MK2^+/+^ and MK2^−/−^ mice were treated with C5a (100 nM) for 2 min and immunofluorescence imaging was performed to detect the localization of p47^phox^ (green). Nuclei were stained with DAPI (blue). The results are representatives of three independent experiments, scale bar is 4 μm.

Phosphorylation of p47^phox^ leads to its membrane translocation that is critical for the formation of the active NADPH oxidase (19). Therefore, we further determined whether MK2 regulated p47^phox^ activation and membrane translocation. As shown in Figures 5A,B, C5a significantly induced p47^phox^ phosphorylation which was abolished after the neutrophils were pre-treated with the p38 inhibitor SB203580 for 15 min. In response to C5a stimulation, phospho-p47^phox^ was evaluated in neutrophils in 1 min, whereas the phospho-p47^phox^ was greatly decreased in neutrophils from MK2^−/−^ mice, compared to that from MK2^+/+^ mice (Figures 5C,D). Furthermore, to test whether MK2 directly phosphorylated p47^phox^, we generated and purified human GST-p47^phox^ (hp47^phox^) and mouse GST-p47^phox^ (mp47^phox^) protein (20), which were then subjected MK2. Phosphorylation of p47^phox^ was evaluated by *in vitro* kinase assays. As showed in Figure 6A, both human p47^phox^ and mouse p47^phox^ protein were phosphorylated by MK2. The results suggested that MK2 could directly phosphorylate p47^phox^.

Because p47^phox^ is responsible for transporting the whole cytosolic complex (p47^phox^-p67^phox^-p40^phox^) to the docking site during NADPH oxidase activation (21), it is possible that MK2 modulates p47^phox^ membrane translocation via phosphorylation of p47^phox^. In naïve neutrophils, the majority of p47^phox^ proteins were located in the cytoplasmic compartment (green) in image a (Figure 6B). Upon treatment with C5a for 2 min, p47^phox^ was translocated to the plasma membrane as shown in image b, which was greatly impaired in MK2^−/−^ neutrophils in image d (Figure 6B). Thus, these data indicated that MK2 directly phosphorylates p47^phox^ and induces membrane translocation of p47^phox^.

### MK2 phosphorylates P47^Phox^ on Ser329

**Figure 7:**
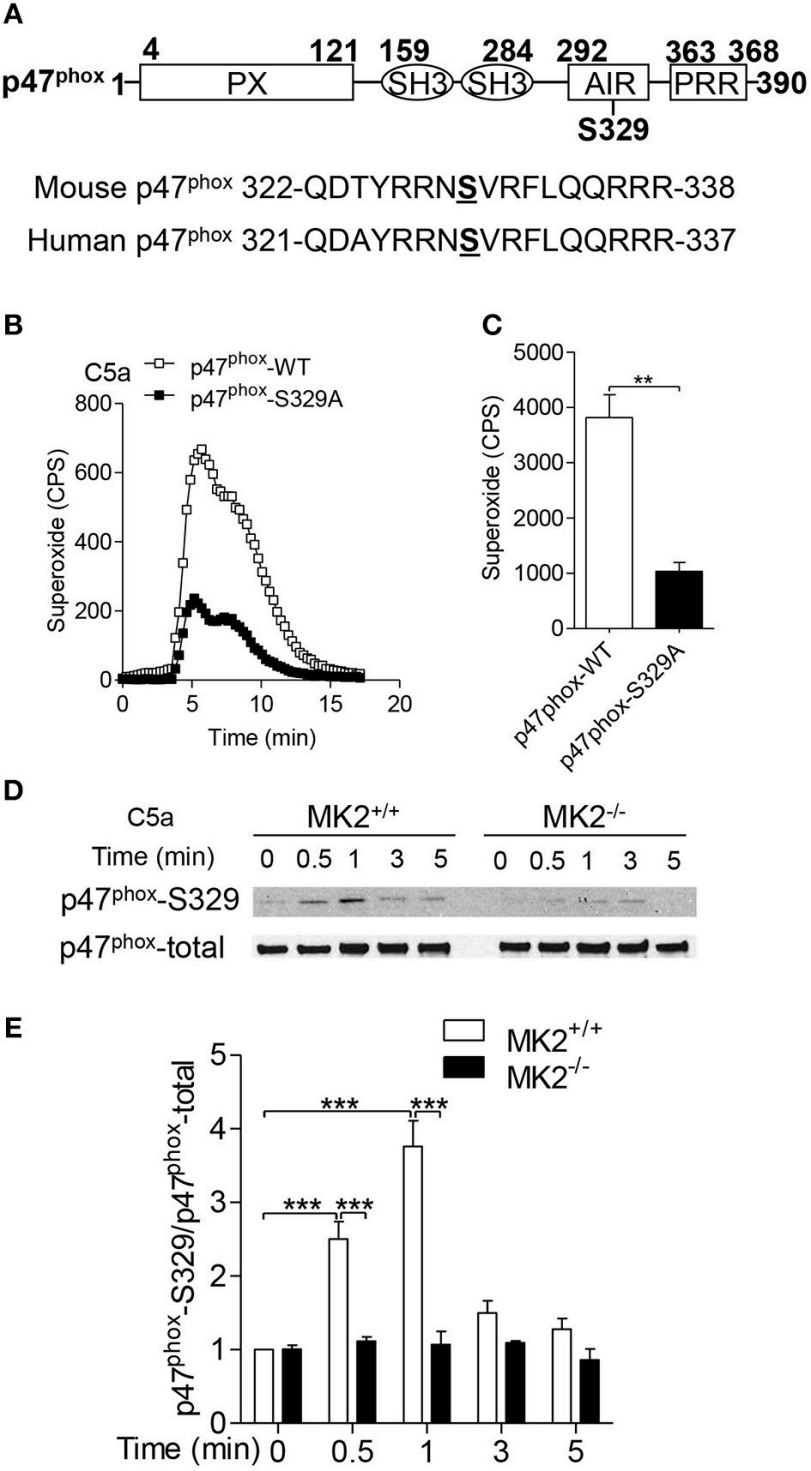
MK2 regulated mouse p47^phox^ at Ser329 residue. **(A)** Alignment of sequence of human and mouse p47^phox^ proteins surrounding the potential MK2 phosphorylation site. **(B)** Neutrophils from *p47*^*phox*−−^^/–^ mice were transiently transfected with full-length p47phox or its S329 mutant for 6 h, and followed challenge with 100 nM of C5a. The production of superoxide production was measured as described. **(C)** The quantitative analysis was performed based on results of B superoxide. **(D)** Neutrophils from MK2^+/+^ and MK2^−−/–^ mice were challenged with 100 nM of C5a for 0.5, 1, 3, and 5 min, the phosphorylation of p47^phox^ (Ser329) was determined by western blotting with anti- p47phox-S329 antibody and p47^phox^-total antibody. **(E)** Densitometry analysis was conducted to determine the relative level of p47^phox^-S329. Data shown are means ± SEM from three independent experiments. **P < 0.01, ***P < 0.001.

The C terminus (between Ser303 and Ser379) of p47^phox^ is a regulatory domain for p47^phox^ activation and membrane translocation (19, 22, 23). To determine the specific phosphorylated site of p47^phox^ by MK2, we compared mouse p47^phox^ with human p47^phox^ and found that Ser329 on mouse p47^phox^ was conservative to human p47^phox^ serine 328 (Figure 7A). To further identify whether Ser329 of p47phox is required for NAPDH oxidase activation, we generated lentivirus with full-length p47^phox^ (p47^phox^-WT) or Ala substitution Ser329 of p47^phox^ (p47^phox^-S329A) mutant and infected them into p47^phox^ deficient neutrophils that were defective for superoxide generation. In response to C5a stimulation, p47^phox^ deficient neutrophils with wide type p47^phox^ (p47^phox^-WT) produced more superoxide than that with p47^phox^-S329A, although neutrophils with p47^phox^-S329A still had capability to generate superoxide (Figures 7B,C). To evaluate whether MK2 modulated p47^phox^ Ser329 phosphorylation, we used antibody specific to phosphorylated p47^phox^ Ser329. Upon treatment with C5a, the phosphorylation of p47^phox^ Ser329 were induced within 0.5 min and reached peak at 1 min (Figures 7D,E). MK2^−/−^ neutrophils displayed an attenuated phosphorylation at Ser329 of p47^phox^ compared to MK2^+/+^ neutrophils. Thus, these data indicate that Ser329 in p47^phox^ sequence is an important regulatory site for NADPH oxidase activation and can be modulated by MK2.

### Myeloid-specific deletion of MK2 alleviated hepatic I/R injury

**Figure 8:**
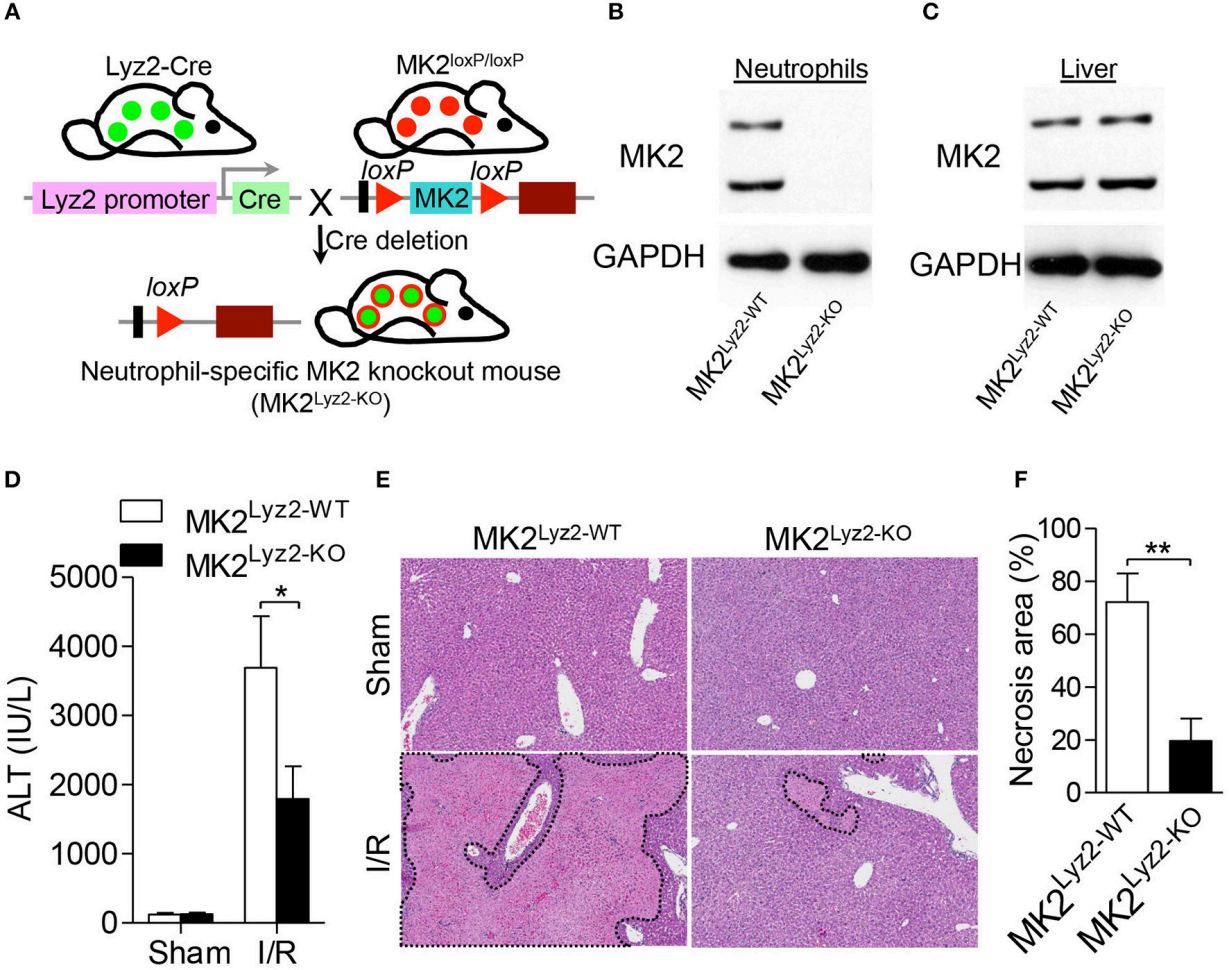
Myeloid-specific deletion of MK2 decreased hepatocellular damage in hepatic I/R injury. **(A)** Schematic representation of the generation of MK2^Lyz2−KO^ and MK2^Lyz2−WT^ mice that MK2^loxP/loxP^ mice were bred with Lysozyme (Lyz2)-Cre transgenic mice. The protein level of MK2 in neutrophils **(B)** and livers **(C)** from MK2^Lyz2−KO^ and MK2^Lyz2−WT^ mice was detected by western blot. MK2^Lyz2−KO^ and MK2^Lyz2−WT^ mice were subjected to hepatic I/R injury with 60 min of ischemia and 16 h of reperfusion. Serum and injury livers were collected for analysis. **(D)** Serum ALT levels were measured. **(E)** Representative H&E stained sections of injury livers were shown. Original magnification × 100. **(F)** The necrosis area was measured by ImageJ software. The results are shown as means ± SEM. *P < 0.05; **P < 0.01, based on 5 mice in each group.

To further prove that the loss of MK2 in neutrophils regulates hepatic I/R injury, we generated myeloid-specific deficiency of MK2 mice (referred to as MK2^Lyz2−KO^) by mating MK2^loxP/loxP^ mice with lysozyme 2-Cre mice (Figure 8A). The expression of MK2 was abolished in neutrophils from MK2^Lyz2−KO^ mice compared with that from MK2^Lyz2−WT^ mice (Figure 8B), which was no significant difference between the liver tissues from MK2^Lyz2−KO^ or MK2^Lyz2−WT^ mice (Figure 8C), suggesting MK2 was deficient in neutrophils. Then, the MK2^Lyz2−KO^ and MK2^Lyz2−WT^ mice were subjected to hepatic I/R. Compared to MK2^Lyz2−WT^ mice, MK2^Lyz2−KO^ mice displayed reduced ALT activities (Figure 8D) and decreased liver necrosis injury (Figures 8E,F) 6 h after hepatic I/R injury. Taken together, these data confirm that MK2 contributes to hepatic I/R injury and modulates neutrophil activation and ROS production.

## Discussion

Hepatic ischemia/reperfusion (I/R) injury is a common pathological process in liver surgery and transplantation, influencing the patient's outcome post-surgery (8). Although p38 MAPK has been implicated in the pathogenesis of hepatic I/R injury (16), the modulatory mechanisms remain elusive. Here, we found that MK2, one of the downstream kinases of p38 MAPK, contributed to hepatic I/R. Genetic MK2 deficiency dramatically limited pathological damage, reduced serum ALT activities, decreased inflammatory cytokine production, and impaired neutrophil infiltration in mice. In *in vitro* study, MK2 deficiency abrogated superoxide production and activation of AKT and p38 MAPK in neutrophils. Furthermore, we identified that NADPH oxidase activation was regulated by MK2 that directly phosphorylated Ser329 of p47^phox^. The myeloid-specific deletion of MK2 mice (MK2^Lyz2−KO^) also displayed a reduced liver injury after I/R injury. Therefore, our findings demonstrated for the first time that down-regulation of MK2 protects against hepatic I/R injury, which could be a novel therapeutic target for I/R injury.

Hepatic I/R injury is closely related to innate immune cell activation and inflammatory processes. Although dendritic cells, natural killer T (NKT) cells, Kupffer cells are all involved in modulation of hepatic I/R injury (4), neutrophils play an nonredundant role in hepatic I/R injury because depletion of neutrophils or neutrophil specific deficiency of MK2 alleviating I/R injury (Figures 2, 8). Coincidentally, recent studies also reveal that neutrophils participate in the hepatic I/R injury. Honda M et al found that formyl-peptide receptor 1 (FPR1)-mediated neutrophil recruitment modulate hepatic I/R injury (24). Infiltrated neutrophils induce hepatocyte death and enhance Kupffer cells to produce inflammatory cytokines by releasing neutrophil extracellular trap (NET) (25). In addition to generation of NET, neutrophils can exacerbate tissue injury though releasing ROS, proteinases and cationic peptides (20). Although we couldn't exclude that MK2 may regulate hepatic I/R injury via modulating Kupffer cells or dendritic cells, our results suggest that MK2-mediated neutrophil activation is involved in hepatic I/R injury.

MK2 is one of the downstream kinases of p38 MAPK that is activated during hepatic I/R injury and regulates liver injury (16). Coxon et al. proved that MK2 regulates human neutrophil activation in p38-dependent and ERK-dependent signal pathways (26). Furthermore, MK2 is involved in neutrophils polarization and chemotaxis and regulates infectious diseases (27, 28). However, a particular role of MK2 in sterile inflammatory responses was not well investigated. In this study, we uncovered the alleviation of liver damage and reduction of liver neutrophil accumulation in genetic MK2 deficiency mice (MK2^−/−^) and myeloid-specific deletion of MK2 mice (MK2^Lyz2−KO^) during haptic I/R injury. These results indicate that MK2 regulates hepatic I/R injury through modulating neutrophil functions. However, it should be noted that, in our *in vivo* data, we only examined that several inflammatory cytokines and chemokines such as IL-6, TNF-α and KC within the liver tissue were decreased in MK2^−/−^ mice. More adhesion molecules such as intercellular adhesion molecule 1 (ICAM-1), integrin need to be further studied to explore the molecular mechanism for the regulatory effect of MK2 in neutrophils chemotaxis, recruitment and transendothelial migration.

Oxidative stress has been considered to be a major part of liver damage induced by I/R injury, and oxygen free radicals are the principal components that lead to hepatocellular necrosis and apoptosis in I/R injury (8). Therefore, antioxidants including superoxide dismutase, catalase, glutathione, vitamin E, and beta-carotene protect against hepatic I/R injury (29). During reperfusion after liver ischemia, neutrophils are a potential source of oxygen free radicals by activation of NADPH oxidase. Here, our data showed that MK2 deficient neutrophils produced less superoxide production in response to C5a or fMLF stimulation but not to PMA. Given that PMA bypasses receptors and directly activates protein kinase C (PKC), MK2 mediates NADPH oxidase activation through specific cell-surface receptors and related molecules, such as phosphoinositide 3-kinase (PI3K), G protein-coupled receptor kinases (GRKs), small GTP-binding proteins, and mitogen-activated protein kinases (MAPK). Consistently, we also found that phosphorylation of AKT and p38 MAPK is reduced in MK2-deficient neutrophils in response to C5a stimulation (Figure 4). Thus, our data suggests that MK2 accentuate superoxide generation and hepatic I/R injury via modulating AKT and p38 MAPK signals.

MK2 is a stress-activated serine/threonine-protein kinase involved in different cellular functions through its kinase activity (9). NADPH oxidase is an inducible electron transport system assembled by several subunits (gp91^phox^, gp22^phox^, p47^phox^, p67^phox^, small GTPase Rac, and p40^phox^) (17). The phosphorylation and membrane translocation of the subunit p47^phox^ is necessary for NADPH oxidase activation and regulation (21, 22). Although several kinases are implicated in regulating the phosphorylation of p47^phox^, the mechanisms and dynamics of p47^phox^-orchestrated NADPH activation haven't been completely revealed. In human neutrophils, phorbol myristate acetate (PMA), and fMLF induce MK2 phosphorylation at Ser334 and further increase NADPH oxidase activation (30). TNF-α, granulocyte-macrophage colony-stimulating factor (GM-CSF), and C5a can induce p47^phox^ phosphorylation through p38 MAPK (31). Thus, p38 MAPK and MK2 are involved in the modulation of NADPH oxidase activation. However, the regulatory mechanisms have not yet been defined. Our *in vitro* kinase assay showed that MK2 can directly regulated the phosphorylation of p47^phox^, thereby reducing neutrophil superoxide production. Moreover, membrane translocation of p47^phox^ was impaired in MK2^−/−^ neutrophils. Our data first demonstrate that MK2 regulates the ROS production in neutrophils through the direct regulatory effect on the phosphorylation of p47^phox^.

Several phosphorylated sites of p47^phox^ are involved in NADPH oxidase activation. The p47^phox^ phosphorylation at Ser345 serves as a point of convergence for different MAPKs to induce priming of ROS production (23). We previously proved that Thr356 of p47^phox^ is a phosphorylation site for p38 MAPK (20). Here, we identified that Ser329 was a new phosphorylation site and could be modulated by MK2. The phosphorylation level of p47^phox^ on Ser329 was abrogated in MK2^−/−^ neutrophils. The p47^phox^ has multiple serine residues ranging from Ser303 to Ser379 (23). Phosphorylation of some serine sites, such as Ser359 or Ser370, is an important initial step to activate p47^phox^ (32, 33). Other residue such as Ser379 can cause inhibition of NADPH oxidase activation (34). Although we identified Ser329 as a new residue that can be regulated by MK2, the synergistic and dynamic effects of these residues on p47^phox^ activation and ROS production should be further explored.

In conclusion, we demonstrated substantial and significant protection against the hepatic I/R injury in genetic MK2 knockout mice and MK2 myeloid-specific deletion mice. In addition, we found MK2 played a vital role in NADPH oxidase activation and ROS production through AKT and p38 signal pathways. Moreover, MK2 regulated NADPH oxidase activation by phosphorylation of p47^phox^ on Ser329 under complement component stimulation. Our findings not only reveal a novel regulatory mechanism for hepatic I/R injury, but also identify a novel therapeutic target for ameliorating hepatic I/R injury in liver transplantation.

## Methods

### Mice

MK2 deficient mice, MK2^loxP/loxP^ mice and lysozyme 2-Cre mice were purchased from The Jackson Laboratory. *MK2*^Lyz2−KO^ Mice were generated by mating MK2^loxP/loxP^ mice with lysozyme 2-Cre mice (35). The p47^phox–/–^ mice was provided by Dr. Steven M Holland (NIH, Bethesda, MD, USA). All mice used in the study were on C57BL/6 background and 8–12 weeks of age. Mice were housed in a climate-controlled room (25°C, 55% humidity and 12 h light/darkness cycles) and all procedures were conducted with the use of protocols approved by the Institutional Animal Care and Use Committee at Shanghai Jiao Tong University.

### Reagents

Phorbol ester phorbol 12-myristate 13-acetate (PMA), *N*-formyl-Met-Leu-Phe (fMLF), C5a and isoluminol were purchased from Sigma-Aldrich (St. Louis, MO, USA). The p38 MAPK inhibitor SB203580 was ordered from Selleck (Houston, TX, USA). Antibodies including Phosphor-Akt, Akt, Phosphor-p38 MAPK, p38 MAPK, MK2, and GAPDH were ordered from Cell Signaling Technology (Danvers, MA, USA). Anti-p47^phox^ antibody was purchased from Santa Cruz Biotechnology and anti-phospho-p47^phox^ (Ser329) antibody was generated by N.J. Compass Biotechnology (Nanjing, China). Other reagents were ordered from Sigma-Aldrich (St. Louis, MO, USA).

### Partial liver ischemia/reperfusion injury (IRI) mouse model

All mice were anesthetized with pentobarbital (5 mg/kg) by intraperitoneal injection. The mouse abdominal cavities were opened with operating scissors. All structures in the portal triad (hepatic artery, portal vein, bile duct) to the left and median liver lobes were occluded with a microvascular clamp (Fine Science Tools) for 60 min; reperfusion was initiated by removal of the clamp. After 6 or 18 h of reperfusion, anesthetized animals were sacrificed, and liver tissue and serum were collected for analysis. Sham-operated groups underwent the same surgical procedure, except that the blood supply to the liver lobes was not interrupted.

### Histopathological analysis

Following euthanasia, representative pieces of ischemic lobes were quickly removed and fixed in ice cold 10% phosphate-buffered formalin for 24 h at 4°C, and then embedded in paraffin. Tissue blocks were sectioned at 4 μm thickness and slices were baked at 60°C for 4 h. After removal of the paraffin by using xylene and a graded ethanol series, the sections were cut to 4-μm-thick sections, stained with hematoxylin and eosin (Beyotime Institute of Biotechnology, China). Following staining, the observer was blinded to treatment group. The slides were viewed under a microscope (Olympus Optical Co. Ltd., Tokyo, Japan).

### Activity assay of ALT

Blood samples were centrifuged for 10 min at 3,000 rpm and serum were collected. The activities of serum alanine aminotransferase (ALT) were measured using the ALT Assay Reagent kit (NJJCBIO, Nanjing, China) according to the manufacturer's instructions with colorimetric evaluation on the microplate reader (FlexStation 3, Molecular Devices, CA, USA).

### Myeloperoxidase activity assay

Frozen mouse liver was homogenized with a Teflon homogenizer in 50 mM phosphate buffer. After centrifugation at 13,000 *g* for 30 min, the cell pellet was resuspended in 1 ml 0.5% hexadecyl trimethylammonium bromide (Sigma-Aldrich, St. Louis, MO, USA), and treated with three cycles of freeze, thaw, and sonication. After centrifugation at 13,000 *g* for 20 min at 4°C, the supernatant was incubated with 16 mM 3,3′,5,5′-tetramethylbenzidine (Sigma-Aldrich, St. Louis, MO, USA) and 15 mM H_2_O_2_, and absorbance at 655 nm was determined. One unit of MPO activity was defined as the change of absorbance of 1.0 per min.

### Superoxide production assays

Mouse polymorphonuclear neutrophils (PMN) were purified from bone marrow cell suspensions as described previously (20). Isolated neutrophils were incubated with 10 μM isoluminol at 37°C for 30 min, and then challenged with PMA (200 ng/mL) for 45 min, fMLF (10 μM) or C5a (100 nM) for 15–60 min. The generation of superoxide was detected by using a Wallac 1420 Multilabel Counter (PerkinElmer, Houston, TX, USA).

### Neutrophil depletion

To deplete neutrophils, mice were intraperitoneally injected with 5 mg/kg (100 μg/mouse) anti-Gr-1 antibody (1A8) that specifically recognized neutrophil surface mark Ly6G (Bio X Cell, West Lebanon, NH, USA) 24 h before ischemia/reperfusion challenge. Isotype-matched IgG of the same amount was used as a negative control.

### Flow cytometric analysis

After treating with anti-Gr-1 antibody for neutrophils deletion, the mice were performed liver ischemia/reperfusion injury (IRI), the percentages of neutrophils were measured using Ly6G-FITC (BD Biosciences, West Lebanon, NH, USA) for flow cytometry. Data was analyzed using FlowJo 7.5 (Tree Star Software).

### Immunofluorescence microscopy

Neutrophils on glass coverslips coated with fibrinogen were stimulated with or without C5a, washed once with ice-cold phosphate-buffered saline (PBS), and then fixed with 3% paraformaldehyde in PBS. The anti-p47^phox^ antibody were used at 1:250 dilution overnight, cells were incubated with a rhodamine green-X-conjugated goat anti-mouse IgG (secondary antibody) at room temperature for 1 h. After washing, the coverslips were mounted on glass slides with the use of ProLong Gold anti-fade reagent with DAPI. Fluorescence images were captured with a confocal microscope with custom software (Leica Micro-systems, CMS GMBH, and LAS AF).

### Quantitative real-time reverse transcription polymerase chain reaction

Total RNA was isolated from liver tissues, and first-strand cDNA was synthesized with Moloney murine leukaemia virus reverse transcriptase (Qiagen, USA). Quantitative real-time polymerase chain reaction (qRT-PCR) was performed with SYBR Green (Invitrogen) in a Bio-Rad real-time PCR detection system with primer sets for IL-6, forward 5′-TAGTCCTTCCTACCCCAATTTCC-3′, reverse 5′- TTGGTCCTTAGCCACTCCTTC-3′; TNF-α, forward 5′-CCCTCACACTCAGATC ATCTTCT-3′, reverse 5′-GCTACGACGTGGGCTACAG-3′; KC, forward 5′-TCCA GAGCTTGAAGGTGTTGCC-3′, reverse 5′-AACCAAGGGAGCTTCAGGGTCA-3′. The expression of each gene was normalized to GAPDH mRNA, and calculated with respect to the baseline control using ΔΔ*Ct* method *via* StepOne 2.0.

### Cytokine production detection

The concentration of TNF-α, IL-6, and KC in supernatants from liver tissues of liver ischemia/reperfusion injury (IRI) mice were evaluated by ELISA, according to manufacturer's instruction (R&D Systems, Minneapolis, MN, USA).

### Kinase assay

*In vitro* kinase assays were performed as previously described by using activate and a recombinant GST-mouse p47^phox^ or GST-human p47^phox^ fusion protein as substrates (20). The samples were analyzed by SDS-PAG autoradiography.

### Western blot analysis

The neutrophils after treatment were washed with DPBS and lysed with RIPA lysis buffer (Thermo Fisher Scientific Inc., Rockford, IL, USA) containing protease inhibitor cocktail (Sigma-Aldrich, St Louis, MO, United States) and phosphatase inhibitor cocktail (Roche Applied Science, Indianapolis, IN, United States). The samples were resolved on SDS-PAGE, and underwent immunoblotting analysis using the indicated antibodies. The protein band intensities were normalized to those of GAPDH. The intensity was quantified by Image J software.

### Lentiviral production and transduction

Full-length p47^phox^ (p47^phox^-WT) and Ala substitution Ser329 of p47^phox^ (p47^phox^-S329A) mutant were cloned into the pLVX-IRES-mCherry vector (Takara CA, USA). HEK293T cells were planted into 90 mm dish at 80% confluence and co-transfected with 10 μg of p47^phox^ plasmid, 7.5 μg of the psPAX2 plasmid and 2.5 μg of pMD2.G plasmid (Addgene, Cambridge, MA) by using polyethyleneimine (Polyscience,USA). Culture supernatants of the HEK293T cells were collected after 72 h and concentrated to 300 μl with a concentration tube. For infection„ p47^phox^ deificient neutrophils were incubated with lentiviral supernatants of different concentrations and Polybrene (Sigma, St. Louis, MO), followed by centrifugation at 2,000 x g for 90 min at 30°C and incubation at 37°C for 4 h. The supernatant was exchanged with fresh complete medium. The cells were stimulated with C5a (100 nM) and harvested for experiment after 6 h.

### Statistical analysis

Each experiment was performed independently for at least 3 times. The results are presented as the mean ± SEM. Statistical significance of differences between groups was analyzed with unpaired Student's *t*-test or one-way ANOVA when more than two groups were compared. Statistical significance was defined as ^*^P < 0.05, ^**^P < 0.01, and ^***^P < 0.001. Analysis and graphing were performed using the Prism software (ver. 5.0; GraphPad, San Diego, CA).

## Author contributions

LS, QW, YN, NC, RW, GW, DZ, and HH performed the experiments. LS, NC, YN, and FQ analyzed the data. LS, RY, and FQ prepared the manuscript.

### Conflict of interest statement

The authors declare that the research was conducted in the absence of any commercial or financial relationships that could be construed as a potential conflict of interest.

## Abbreviations

I/R injury: Hepatic ischemia/reperfusion injury
ROS: reactive oxygen species
MK2: MAPK-activating protein kinase 2
ALT: alanine amino transferase
AST: aspartate amino transferase
PMA: phorbol ester phorbol 12-myristate 13-acetate
fMLF: N-formyl-Met-Leu-Phe
MPO: Myeloperoxidase
NADPH oxidase: nicotinamide adenine dinucleotide phosphate oxidase
PMN: polymorphonuclear neutrophils
KCs: Kupffer cells.
